# Semitendinosus muscle morphology in relation to surface electrode placement in anterior cruciate ligament reconstructed and contralateral legs

**DOI:** 10.3389/fspor.2022.959966

**Published:** 2022-11-04

**Authors:** Adam Kositsky, Rod S. Barrett, William du Moulin, Laura E. Diamond, David J. Saxby

**Affiliations:** ^1^Griffith Centre of Biomedical and Rehabilitation Engineering (GCORE), Menzies Health Institute Queensland, Griffith University, Gold Coast, QLD, Australia; ^2^Department of Applied Physics, University of Eastern Finland, Kuopio, Finland

**Keywords:** electromyography, graft, hamstrings, SENIAM, ultrasound

## Abstract

The semitendinosus tendon is commonly harvested as graft tissue for anterior cruciate ligament reconstruction (ACLR). Although the semitendinosus tendon can regenerate following harvesting, ACLR results in substantial reductions in semitendinosus muscle size and length, potentially complicating electrode placement for electromyography. The purpose of this study was to assess whether the most commonly used electrode placement [recommended by the “Surface Electromyography for Non-Invasive Assessment of Muscles” (SENIAM) project] is appropriate for measuring semitendinosus electromyograms after ACLR. In nine participants (unilateral ACLR with a semitendinosus graft), B-mode ultrasonography was used to bilaterally determine (i) the semitendinosus muscle-tendon junction position and the state of tendon regeneration (latter for the ACLR leg only) and (ii) the anatomical cross-sectional area (ACSA) of the semitendinosus muscle at the SENIAM-recommended electrode placement site at rest and during isometric maximal voluntary contraction (MVC) at two knee joint angles. Depending on the contraction state and joint angle, the semitendinosus muscle had retracted past the recommended placement site in 33–78% of ACLR legs, but not in any contralateral legs. The ACSA of semitendinosus was smaller both at rest and MVC in the ACLR compared to contralateral leg. The ACSA for both legs decreased at MVC compared to rest and at deep compared to shallow knee flexion angles, likely due to sliding of the muscle under the skin. These results suggest SENIAM guidelines are likely unsuitable for recording surface electromyograms from the semitendinosus muscle after tendon harvesting for ACLR as the muscle of interest may not be within the electrode detection volume.

## Introduction

Because of the biomechanical properties of the tendon and graft and the small surgical incision size that circumvents affecting the knee extensor mechanism (1), the whole distal semitendinosus (ST) tendon (sometimes including the distal gracilis tendon) is widely used worldwide for anterior cruciate ligament reconstruction (ACLR) (2), a debilitating injury and treatment that is increasingly common (3). After ST tendon harvesting for ACLR, long-term morphological changes often persist; ST muscle volume and maximal anatomical cross-sectional area (ACSA) are ~25–45% and ~20–30% smaller, respectively, and the ST is ~4–10 cm shorter when assessed by either change in muscle length or proximal shift in the distal muscle–tendon junction (MTJ) position (4–13). Despite these morphological changes, the harvested ST tendon has potential for regeneration and bony reattachment below the knee joint line (14), which could result in restoration of some level of function to a muscle that is important for high-velocity athletic tasks such as sprinting (15, 16).

Electromyography (EMG) can be used to indirectly assess some aspects of muscle function, such as muscle activity amplitudes, patterns, and temporal relationships. However, the specificity of surface EMG recordings depends on careful placement of the electrode(s) over the muscle(s) of interest. Many studies continue to use electrode placement recommendations from the “Surface Electromyography for Non-Invasive Assessment of Muscles” (SENIAM) project (17). Accordingly, numerous studies including participants post-ACLR with an ST graft placed electrodes halfway between the ischial tuberosity and the medial tibial epicondyle (as per SENIAM guidelines) to measure ST muscle activity (18–28), as a surrogate of all medial hamstring activity (29–35), and to drive EMG-based neuromusculoskeletal models (36, 37). Other studies placed bipolar electrodes more distally on the thigh (38, 39). However, none of these studies reported using ultrasound guidance for electrode placement, suggesting the alterations in muscle–tendon unit morphology (i.e., what is beneath the electrode) may not have been fully considered. Given substantial muscle shortening following ACLR, the ST muscle may not always be present under electrodes placed according to recommendations for healthy legs. Furthermore, moving from shallow to deep knee flexion angles and/or ST muscle activation could result in further ST muscle shortening (40, 41), potentially past the SENIAM-recommended location.

Therefore, the purpose of this study was to assess whether the commonly used surface electrode placement location would be suitable for EMG measures of ST in individuals post-ACLR. We investigated the bilateral presence and ACSA of the ST muscle at 50% of the distance from the ischial tuberosity to the medial tibial epicondyle at rest and during maximal isometric contractions at two knee joint angles (15° and 90°). We hypothesized that the electrode location for the ST as set out by the SENIAM guidelines would not be suitable for ACLR legs, owing to a significantly smaller length and size of the ST muscle, and that the knee flexion joint angle and contraction state would influence the presence of the ST muscle in ACLR legs and reduce the ACSA of ST in both legs.

## Methods

### Participants

A total of 10 individuals who underwent unilateral ACLR using an ipsilateral distal ST tendon autograft volunteered to participate in the study. All ACLRs were performed by one surgeon, and the ACLR procedure is described in a companion paper from this data collection (42). Due to poor-quality ultrasound recordings from one participant, data are reported for nine participants (six women; age: 27.2 ± 5.2 years; height: 170.3 ± 9.6 cm; mass: 71.4 ± 13.7 kg). The participants were 436 ± 85 days post-surgery, representing a time frame reflective of return to physical activity. Potential participants were excluded from the study if they had ACLR more than 6 months after ACL injury, gracilis tendon harvest in ACLR, previously sustained other major knee injuries, neurological disorders, or physician recommendations to avoid undergoing a magnetic resonance imaging scan. The participants were requested to refrain from strenuous exercise commencing 24 h prior to the investigation. The study was approved by the Griffith University Human Research Ethics Committee (2018/839) in accordance with the Declaration of Helsinki. All participants provided written informed consent prior to any involvement in the study.

### Experimental design

The participants attended a laboratory session where B-mode ultrasound was used to (i) identify any presence of ST tendon regeneration in the ACLR leg and the bilateral location of the distal MTJ at rest, and (ii) measure the ACSA of the ST muscle at the recommended electrode location in ACLR and contralateral legs during rest and at maximum isometric knee flexion contractions of 15° and 90° of knee joint flexion (0° = full extension). These joint angles were chosen due to their relevance for assessing potential neural influences on knee flexion strength after ACLR; weakness is consistently documented at deep knee flexion angles, such as 90°, but not always at shallow angles (6, 10). All measurements were obtained bilaterally.

### Assessment of ST tendon regeneration and distal MTJ position

**Figure 1:**
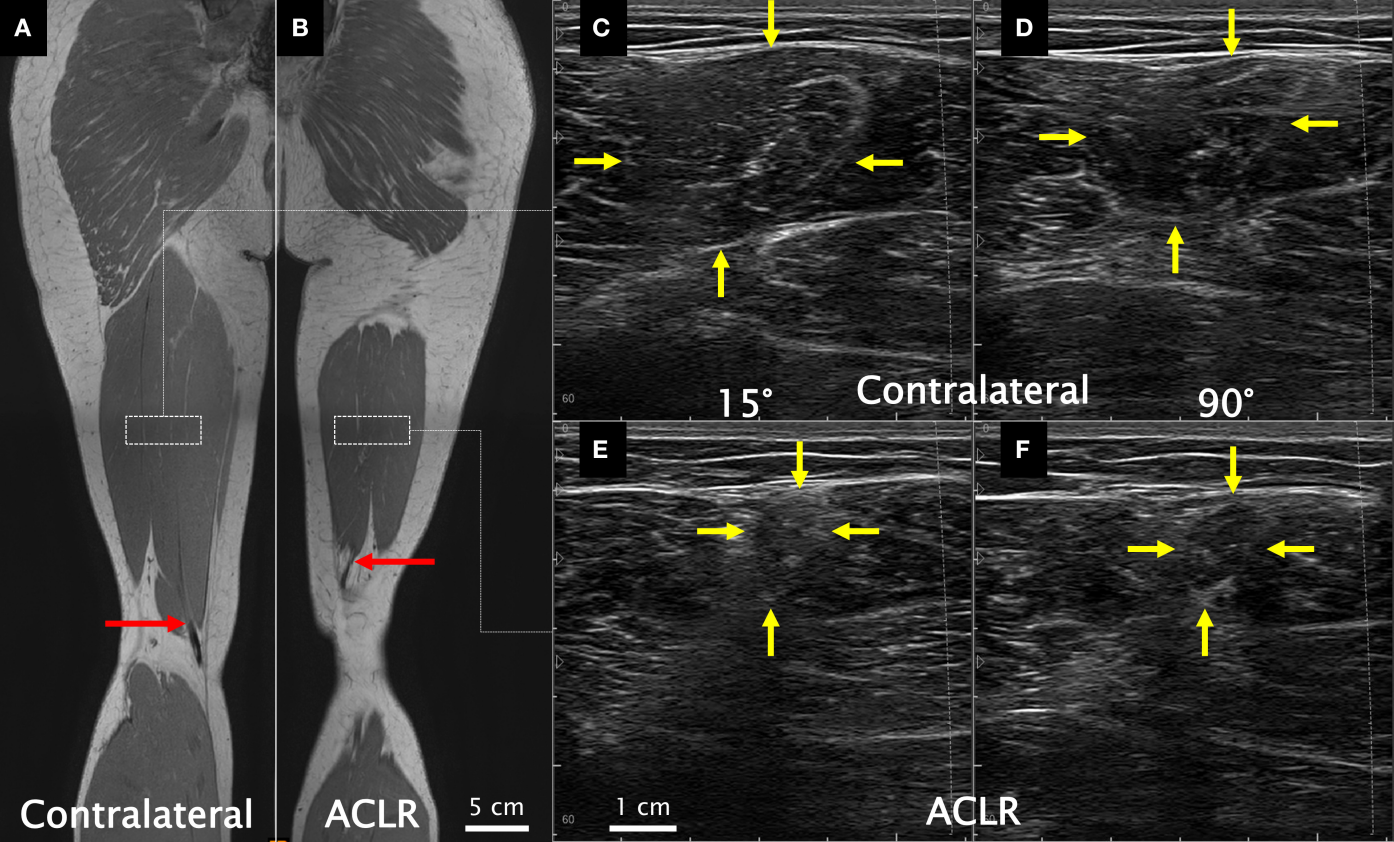
Coronal view from the in-phase sequence of a T_1_ Dixon magnetic resonance image (MRI) series acquired supine (used here only for illustrative purposes) of the contralateral **(A)** and the anterior cruciate ligament reconstructed (ACLR) **(B)** legs. Red arrows illustrate the end of the muscle–tendon junction (beginning of the free tendon), which was quantitatively assessed from ultrasound. Dashed white rectangles approximate the recommended proximodistal electrode placement according to SENIAM guidelines. Resting ultrasound images of the contralateral leg at 15° **(C)** and 90° **(D)** of knee joint flexion and of the ACLR leg at 15° **(E)** and 90° **(F)** of knee joint flexion. Yellow arrows indicate the semitendinosus muscle borders. Note that the MRI images are not from the same coronal slice for both legs and do not capture the full length of the semitendinosus muscle within a given slice (slices for this figure were chosen independently for each leg to best visualize the distal semitendinosus muscle–tendon junction). Also note that the ultrasound images were acquired so all images contained the same left-to-right orientation. All images are from the same participant.

With the participants positioned prone (hip and knee neutral), the lower posterior thigh was scanned to detect any presence of regenerated ST tendon tissue using a 30 mm linear ultrasound transducer (L18-7H30-A5; ArtUS, Telemed, Vilnius, Lithuania) operating at 18 MHz (depth: 30 mm). Subsequently, the posterior thigh was scanned to locate the most distal aspect of the distal ST MTJ (i.e., the beginning of the free tendon where the tendon was regenerated, or the distal muscle stump if the tendon was not regenerated). The location of the distal ST MTJ was marked on the skin surface with indelible ink. The distance from the distal MTJ to the popliteal crease was recorded with a flexible tape measure. As this measurement has been shown to be reliable (43), the between-leg difference was used as a clinically viable estimate of longitudinal ST muscle shortening. A qualitative depiction of the proximal shift in the distal ST MTJ position is shown in Figures 1A,B.

### Assessment of ST ACSA at the recommended electrode location

After a warm-up of 5 min on a cycle ergometer at a freely chosen pace, the participants performed isometric knee flexion maximal voluntary contractions (MVCs) on an isokinetic dynamometer (System 4 Pro, Biodex Medical Systems Inc., Shirley, NY, USA) at 15° and 90° of knee joint flexion. The participants also performed MVCs at 45° and 60° of knee flexion as part of larger data collection, but ultrasound images were not obtained at those joint angles. All trials were performed with the participants positioned prone, strapped to the bed at the waist level, and the ankle locked in a neutral position with an ankle cast (Aircast AirSelect Short, DJO Global Inc., Lewisville, TX, USA). Following a warm-up series of submaximal isometric knee flexion contractions at volitional intensity, a minimum of three MVCs of ~3- to 5-s duration were performed with 1-min rest between efforts. Further MVCs were performed if the third, or subsequent, efforts were ≥ 5% in the maximal torque magnitude than previous efforts (44). Note that the torque data are not reported here. The order in which legs and knee joint angles were tested was randomized using a random number generator (MATLAB version R2018b, MathWorks, Natick, MA, USA). A 2-min rest was given between knee joint angle changes and at least 10-min rest was given between legs.

Transverse ultrasound images were obtained by a single experienced investigator (AK) at the midpoint of the distance between the ischial tuberosity and the medial tibial epicondyle in accordance with the SENIAM guidelines for ST (17) with a 60 mm linear transducer (L12-5N60-A2; ArtUS, Telemed, Vilnius, Lithuania) operating at 8.5 MHz (depth: 60 mm). After marking the location on the skin surface with indelible ink, visibility of the ST muscle underneath the ink marking (i.e., if the muscle had or had not shortened past the scan location) was noted. If the ST muscle could be visualized, the ultrasound transducer was held over the ink marking with minimal compression, and cine loop images were recorded at 30 Hz during MVCs, including approximately 3 s prior to contraction (Figures 1C–F). The transducer was replaced over the skin for each effort, and no noticeable shifting of the ultrasound transducer relative to the ink marking was observed during MVCs. The ACSA of ST from one trial was measured using the polygon tool in ImageJ (Version 1.52a, National Institutes of Health, Bethesda, MD, USA). Rest ACSA values were obtained from one image prior to the onset of contraction, and MVC ACSA values from an image during the plateau phase of the isometric contraction. If the muscle had shortened past the scanning location, the value was recorded as 0 cm^2^. Recordings were re-analyzed 6 months later by the same investigator (AK) to determine intra-rater reliability, which resulted in intra-class correlation coefficients (A,1) and 95% confidence intervals of 0.973 (0.917–0.989) at rest and 0.972 (0.928–0.988) during MVCs.

### Statistical analyses

The effect of leg (contralateral, ACLR) on the distal MTJ position was assessed using a paired *t*-test. All data were normally distributed, except the following: ACSA on the ACLR leg at MVC at 15°, resting ACSA on the contralateral leg at 90°, and ACSA at MVC at 90° on both legs. To bypass distribution assumptions (45), a full-factorial linear mixed model with restricted maximum likelihood estimation was used to assess the effect of leg, contraction state (rest, MVC), and knee joint angle (15°, 90°) on ST ACSA. Leg, contraction state, and knee joint angle were set as fixed factors and repeated measures (first-order autoregressive covariance structure), with participants and their intercepts as random factors. The degrees of freedom were calculated using the Satterthwaite approximation. When significant interactions were detected, *post hoc* Bonferroni tests were conducted to identify significant differences between legs at each contraction state or joint angle condition. Partial eta squared ($\eta_{\text{p}}^{2}$) was estimated and adjusted for bias (46). All hypothesis tests were performed using SPSS (version 27, SPSS Inc., Chicago, IL, USA) with statistical significance set at *p* < 0.05. Data are presented as mean ± one standard deviation.

## Results

Of the nine participants, six had ST tendon regeneration in their ACLR leg. The distance from the distal MTJ to the popliteal crease was significantly larger in the ACLR (10.5 ± 4.4 cm) than the contralateral (5.3 ± 1.8 cm) leg (net shortening [mean difference]: 5.2 ± 4.0 cm; *t* = −3.893; *p* = 0.005; $\eta_{\text{p}}^{2}$ = 0.611). Consequently, the ST muscle was not visible at rest in three ACLR legs at 15° and in four at 90° knee flexion. In a further three ACLR legs at 90° knee flexion, the ST muscle was visible only at rest, leading to no visible ST muscle in seven ACLR legs during MVC at 90° knee flexion.

**Figure 2:**
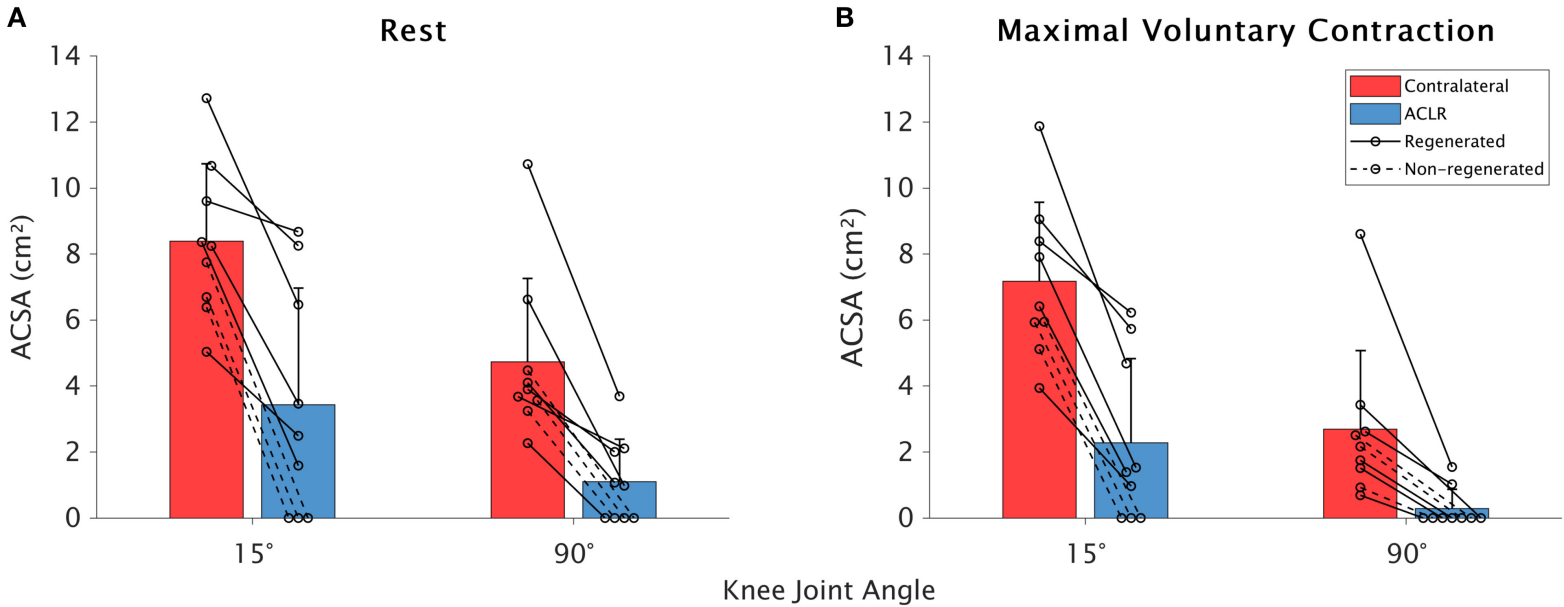
Means and standard deviations for between-leg differences in the semitendinosus anatomical cross-sectional area (ACSA) at rest **(A)** and during maximal voluntary contraction **(B)** in the contralateral (red) and anterior cruciate ligament (ACLR; blue) legs at 15 and 90° of knee joint flexion. Data from participants whose tendon was regenerated on the ACLR side are shown with a solid line, while those without tendon regeneration are denoted with a dashed line.

The ST ACSA data are shown in Figure 2. Significant effects on ST ACSA were detected for leg [*F*_(1,13.43)_ = 65.11; contralateral: 5.7 ± 3.2 cm^2^; ACLR: 1.8 ± 2.5 cm^2^; *p* < 0.001; $\eta_{\text{p}}^{2}$ = 0.816], knee joint angle [*F*_(1,50.55)_ = 82.47; 15°: 5.3 ± 3.7 cm^2^; 90°: 2.2 ± 2.5 cm^2^; *p* < 0.001; $\eta_{\text{p}}^{2}$ = 0.612], and contraction state [*F*_(1,45.30)_ = 28.69; rest: 4.4 ± 3.6 cm^2^; MVC: 3.1 ± 3.3 cm^2^; *p* < 0.001; $\eta_{\text{p}}^{2}$ = 0.374]. The leg–angle interaction was significant [*F*_(1,33.50)_ = 5.48; *p* = 0.025; $\eta_{\text{p}}^{2}$ = 0.115], while there were no significant leg–contraction [*F*_(1,54.44)_ = 1.40; *p* = 0.243; $\eta_{\text{p}}^{2}$ = 0.007], angle–contraction [*F*_(1,49.49)_ = 0.16; *p* = 0.687; $\eta_{\text{p}}^{2}$ = −0.017], or leg-angle-contraction [*F*_(1,53.32)_ = 1.04; *p* = 0.312; $\eta_{\text{p}}^{2}$ = 0.001] interactions. *Post hoc* tests revealed that compared with the contralateral leg, ST ACSA on the ACLR leg was significantly smaller at 15° (*p* < 0.001; $\eta_{\text{p}}^{2}$ = 0.744) and 90° (*p* < 0.001; $\eta_{\text{p}}^{2}$ = 0.516). Furthermore, ST ACSA was significantly smaller at 90° than at 15° for both the contralateral (*p* < 0.001; $\eta_{\text{p}}^{2}$ = 0.580) and ACLR (*p* < 0.001; $\eta_{\text{p}}^{2}$ = 0.273) legs.

## Discussion

This study investigated the presence and size of the ST muscle at the ST EMG electrode location as recommended by SENIAM guidelines (17) bilaterally in individuals with ACLR at rest and during knee flexion MVCs at two knee joint angles. Agreeing with our first hypothesis, the ST muscle at the SENIAM-recommended location was, when present, smaller on the ACLR than the contralateral leg. Furthermore, consistent with our second hypothesis, voluntary muscle contraction and knee flexion angle influenced the presence and size of the ST muscle at the SENIAM-recommended location. Overall, recording surface electromyograms at the SENIAM-recommended position may not be suitable for high-fidelity recordings of ST in ACLR legs, and caution may also be warranted in non-ACLR legs due to the morphological changes induced by altering knee joint angles and contraction states.

### Effects of muscle atrophy on electrode placement at the recommended location

The proximal shift of the distal ST MTJ (5.2 ± 4.0 cm) in the ACLR leg was greater than both the minimal detectable change [1.26 cm; (43)] and the normal between-leg variation in ST muscle length [<1 cm; (13)], and was within the range of ST muscle shortening after ACLR (mean of ~4–10 cm), as reported in previous studies (4–13). Consequently, the ST muscle was not visible at the imaging location for 33–78% of ACLR legs, depending on the knee joint angle and contraction state. At a more proximal imaging location, Morris et al. (47) did not observe the ST in two of 15 ACLR legs, but in that study ST was seemingly present in all legs at approximately the same imaging location used in our study. The larger proportion of ACLR legs without presence of ST in the current study, particularly at 90° and during MVC compared with 15° and rest, respectively, is due to further proximal muscle shortening that occurs with joint motion in a shortening direction (48) and muscle activation (49). As a result, in some individuals, even those with ST tendon regeneration in their ACLR leg, bipolar EMG electrodes placed according to SENIAM guidelines would not be atop the ST muscle. In other participants, such electrode placement would be close to the distal ST tendon, which, irrespective of other morphological issues, affects EMG measurements (17, 50). Thus, it is imperative for future studies using EMG for ST after ACLR to consider muscle morphology when positioning electrodes.

As expected, ST ACSA on the ACLR leg was significantly smaller than that on the contralateral side in all assessed conditions. Nonetheless, the mean between-leg difference in resting ST ACSA (59–77%) was substantially larger than that previously reported for maximal ACSA [~20–30%; (4, 11, 12)]. As shown in Figure 1, the entire ST ACSA fit within the ultrasound field of view for all participants, whereas panoramic (extended field of view) images are typically required at the site of maximal ACSA, generally located at or above halfway between the greater trochanter and the lateral knee joint (43, 51). Conversely, SENIAM guidelines recommend placing the electrode more inferiorly at the midpoint from the ischial tuberosity to medial tibial epicondyle, a position distal to the location of maximal ST ACSA, and even more distal on ACLR legs due to muscle shortening. Therefore, the large between-leg ACSA difference results from comparing measures obtained from the same absolute location along the thigh but different locations relative to ST muscle length, which is consistent with recent findings of larger relative differences in ST ACSA at distal than at more proximal thigh locations (47). As the adjacent knee flexor synergists are near their maximal size at the location for ST as set out by the SENIAM guidelines (43, 51) and these synergists generally do not undergo volumetric atrophy after ACLR (4, 6, 11, 12), the consequence is a smaller ST muscle (if not fully retracted past) sandwiched between large synergistic muscles. To facilitate morphology measures, we obtained images at the SENIAM-defined proximodistal location but maneuvered the transducer to center on the ST, which overlooks potential additional errors from poor mediolateral electrode positioning (52). Thus, bipolar surface EMG recorded from this location is likely to be contaminated by the adjacent muscles (i.e., cross-talk), even if the electrode is centered over the ST, which may not necessarily occur (i.e., slight deviations in the mediolateral position or the muscle retracted past the location). As such, signals recorded *via* electrodes placed on the posterior thigh according to SENIAM guidelines may not accurately reflect ST muscle activity in ACLR legs (where the ST was used as graft donor), which may not necessarily be inferred from synergist muscles because myoelectric activity amplitudes and patterns differ between hamstring muscles (53–59). Therefore, future work is needed to clarify if signals acquired from a more specific location over the ST may result in different interpretations of ST myoelectric activity post-ACLR compared with data obtained from electrodes placed according to SENIAM guidelines.

### Effects of joint movement and MVC on ST ACSA at the recommended electrode location

Varying knee flexion angle and activating the knee flexors induced changes in ST ACSA in both legs, likely due to the constant imaging location along the thigh. The ACSA of ST measured at standardized, distal locations along the thigh appears to become smaller with knee flexion (60). Although Raiteri et al. (49) found the ACSA of tibialis anterior increased with activation across almost the entire muscle length, comparisons at the same location along the leg were not made. By contrast, during elbow flexion contractions, Akagi et al. (61) observed the ACSA of the elbow flexors to increase proximal, and decrease distal, relative to the location of the resting maximal ACSA when obtaining measures at constant positions along the upper arm. As muscles should bulge to maintain a constant volume during contraction (49, 62), the smaller ACSA measured at a constant location along the thigh indicates measures in the two conditions (15 vs. 90°, rest vs. MVC) are not obtained from the same portion of the muscle. Shortening of ST in the proximal direction during contraction or when the knee is flexed to 90° shifts the already distal imaging location in the current study to an even more distal portion of the ST muscle and leads to muscle shortening past the imaging location in some ACLR individuals (see Supplementary Video). Hence, as electrodes are typically placed under conditions that differ from the task(s) participants perform during the experimental procedure, it is important to consider muscle morphology during the contraction state and posture conditions in which recordings from surface EMG may be used.

### Additional morphological factors to consider for electrode placement

In addition to the practical implications described previously resulting from morphological changes in the ACLR leg (due to surgical intervention) or in both legs (due to joint angle change and/or voluntary activation), the general morphology of the ST also needs to be considered when placing surface EMG electrodes. The ST contains a tendinous inscription separating the muscle into two neuromuscular compartments with separate nerve branch innervations (63). At the SENIAM-recommended location, recordings are generally obtained from the distal ST compartment. As most ST muscle fascicles attach directly onto the tendinous inscription (63), blindly (i.e., without ultrasound guidance) placing the electrode proximally to the SENIAM-recommended location may lead to an electrode being placed atop, or on either side of, this inscription, and thus potentially over different neuromuscular compartments of the muscle. Although ultrasound guidance should be used to ensure electrodes are placed atop the ST throughout all testing conditions, bipolar surface electrode placement may be further complicated by the 3 cm shift in the ST innervation zone when the knee is moved through its entire range of motion (50), which is likely related to the >6 cm ST muscle length change from full extension to 90° of knee joint flexion found in cadavers (64, 65). The latter agrees with the rationale for the change in ACSA between joint angles found for both legs in the present study. As the location of the innervation zone with respect to the recording electrode affects the amplitude and spectral properties of the EMG signal, differences in bipolar surface EMG measures of the ST taken at different knee joint angles may have anatomical, rather than physiological, explanations (50). Multi-channel arrays, which may be able to account for sliding of the muscle under the skin (50, 66), may be more advantageous for recording electromyograms of the ST. However, multi-channel arrays may be less accessible and/or not suitable for complex data collections involving multiple muscle groups. Therefore, despite similar mechanical functions between ST compartments (67), future studies should confirm if (i) similar ACSA changes and movement under the skin also occur in the proximal compartment of the ST, and (ii) if recording from the proximal compartment would affect the interpretation of ST muscle activity during functional tasks as differences in EMG amplitudes have previously been reported between ST compartments at some submaximal MVC levels in humans (68), as well as during some locomotor tasks in cats (69).

### Limitations

Study limitations resulting from an absence of concurrent EMG and ultrasound measurements are twofold. First, rather than directly comparing EMG measures obtained from different locations and muscles, we adopted a morphological approach, and the results demonstrate the recommended location for electrodes, particularly following ST ACLR, would either expose the recordings to large amounts of cross-talk or not record from the ST at all. Second, we do not have an EMG recording to confirm muscle relaxation in the resting condition. However, we instructed the participants to remain fully relaxed prior to contraction and did not detect fluctuations in baseline torque prior to contraction or any muscle contraction during the resting part of the analyzed cine loop images. Next, we obtained “control” measures only from the contralateral leg. However, as hamstring morphology on the contralateral leg does not change after ACLR with an ipsilateral ST graft (4) and is comparable with legs of control group participants (47), the results obtained from the contralateral leg are likely representative of a pre-injury state. Furthermore, despite the relatively small sample size, this study was conceived based on similar observations during pilot tests performed on participants with ACLR performed by surgeons from different countries. Thus, these results are likely generalizable across the ST-tendon ACLR demographic. Finally, we only tested knee flexion in the prone position, and it remains unknown how regional ST morphology in static and dynamic states may change with isolated or concomitant hip flexion.

## Conclusion

Due to muscle shortening and radial atrophy, using the SENIAM-recommended electrode placement to measure ST muscle activity after ACLR with an ST graft is unlikely to result in high-fidelity recordings. The single-site ACSA of ST changes with contraction state and joint angle, likely due to substantial sliding of the muscle underneath the skin, which may have implications for obtaining and comparing EMG recordings across different conditions. Therefore, using the SENIAM-recommended location does not appear to be suitable for recording bipolar surface electromyograms of ST following ACLR with an ST graft, and caution may be warranted for healthy legs.

## Data availability statement

The raw data supporting the conclusions of this article will be made available by the authors upon request.

## Ethics statement

The studies involving human participants were reviewed and approved by Griffith University Human Research Ethics Committee (2018/839). The patients/participants provided their written informed consent to participate in this study.

## Author contributions

AK, LED, and DJS contributed to the study conception and design. AK and WdM collected the data. AK analyzed the data and drafted the manuscript. RSB and DJS assisted with data interpretation and initial manuscript drafting. All authors have read and approved the final version of the manuscript, and agree with the order of presentation of the authors.

## Funding

This work was supported by a Griffith University Postgraduate Research Scholarship.

## Conflict of interest

The authors declare that the research was conducted in the absence of any commercial or financial relationships that could be construed as a potential conflict of interest.

## Publisher's note

All claims expressed in this article are solely those of the authors and do not necessarily represent those of their affiliated organizations, or those of the publisher, the editors and the reviewers. Any product that may be evaluated in this article, or claim that may be made by its manufacturer, is not guaranteed or endorsed by the publisher.
